# Artificial intelligence approach for the analysis of placebo-controlled clinical trials in major depressive disorders accounting for individual propensity to respond to placebo

**DOI:** 10.1038/s41398-023-02443-0

**Published:** 2023-04-29

**Authors:** Roberto Gomeni, Françoise Bressolle-Gomeni, Maurizio Fava

**Affiliations:** 1 Pharmacometrica, La Fouillade, France; 2 Department of Psychiatry, Massachusetts General Hospital, and Harvard Medical School, Boston, MA USA

**Keywords:** Diseases, Depression

## Abstract

Treatment effect in clinical trials for major depressive disorders (RCT) can be viewed as the resultant of treatment specific and non-specific effects. Baseline individual propensity to respond non-specifically to any treatment or intervention can be considered as a major non-specific confounding effect. The greater is the baseline propensity, the lower will be the chance to detect any treatment-specific effect. The statistical methodologies currently applied for analyzing RCTs doesn’t account for potential unbalance in the allocation of subjects to treatment arms due to heterogenous distributions of propensity. Hence, the groups to be compared may be imbalanced, and thus incomparable. Propensity weighting methodology was used to reduce baseline imbalances between arms. A randomized, double-blind, placebo controlled, three arms, parallel group, 8-week, fixed-dose study to evaluate efficacy of paroxetine CR 12.5 and 25 mg/day is presented as a cases study. An artificial intelligence model was developed to predict placebo response at week 8 in subjects assigned to placebo arm using changes from screening to baseline of individual Hamilton Depression Rating Scale items. This model was used to predict the probability to respond to placebo in each subject. The inverse of the probability was used as weight in the mixed-effects model applied to assess treatment effect. The analysis with and without propensity weight indicated that the weighted analysis provided an estimate of treatment effect and effect-size about twice larger than the non-weighted analysis. Propensity weighting provides an unbiased strategy to account for heterogeneous and uncontrolled placebo effect making patients’ data comparable across treatment arms.

## Introduction

The unpredictable high placebo response rate is one of the major factor associated with the failure of randomized clinical trials in psychiatric disorders, neuropathic pain, cancer pain, multiple sclerosis, Parkinson disease, and more [1]. Further, evidence has shown that placebo responses were increasing over time in some indications [2–5], without a commensurate increase in response to active treatment; thus, the necessity to learn how to control and mitigate the placebo response, in the context of randomized placebo controlled clinical trials (RCTs), has become increasingly vital.

Several methods to control the placebo response without undermining the observed response to active treatment have been attempted in clinical trials, such as exclusion of placebo responders during placebo lead-in periods [6], alternative study designs such as sequential parallel comparative designs (SPCD) [7, 8], and various methods for detecting and controlling non-plausible placebo response rates at specific clinical trial sites such as the band-pass methodology [9, 10]. All these methods attempt to control the impact of an excessively high placebo response by identifying and removing from the analysis the subjects in the recruitment sites with excessively high placebo response. However, none of these methods propose statistical criteria for assessing the treatment effect (TE) conditional to this propensity to respond to placebo, preserving the integrity of the data collected and without removing any subject from the analyses.

In RCTs, the placebo response usually refers to the degree of clinical improvement reported by patients assigned to the placebo arm, while the placebo effect (PE) represents an improvement in clinical outcomes due to the expectancies of positive treatment or intervention [11]. PE can be defined as the clinical improvement associated with the patient’s interactions with the clinician, the information they received with regard to their condition and treatment, the therapeutic care conditions and to the overall expectation of a clinical benefit of a treatment or intervention [12]. Expectation, usually defined as the subject’s belief about the potential effect of a treatment, was identified as a major non-specific effect that influences the individual level of PE [13].On the basis of these considerations, the effect of a treatment can be viewed as the resultant of two components: the treatment specific and the treatment non-specific effects. The individual propensity to respond to any treatment or intervention assessed at baseline can be considered as a major non-specific prognostic and confounding effect. The larger is the baseline propensity to respond to non-specific treatment, the lower will be the chance to detect any treatment-specific effect. In the context of RCTs, it has been demonstrated that, as the PE increases, the difference between the placebo and active arm decreases, reducing the likelihood that the trial will meet statistical significance of the primary endpoint [4, 14, 15]. The individual baseline propensity to respond to placebo is associated with the individual expectations, varying from individuals to individuals, and is not controlled by the currently standard randomization process as the individual propensity value is unknown. In RCTs, subjects are assigned to the treatment arms at random. As a consequence, potential confounders are expected to be randomly distributed over the arms, which make the arms comparable or balanced. Remaining differences between randomized arms, such as the individual baseline propensity to respond to placebo, are treated as a function of chance. Hence, the groups to be compared may be imbalanced, and thus incomparable due to baseline differences that are not recognized.

The propensity weighting methodology was proposed as a novel method of causal inference that aims at reducing imbalances between arms [16–18]. This technique is based on the calculation of propensity, defined as the individuals’ probability of showing PE, given observed baseline and pre-randomization response [19]. Propensity scores allow researchers to create balance between treatment and comparison arms based on observed confounders such as the PE [20]. The higher is the individual propensity to show a PE, the lower will be the probability to detect a TE. This because the observed signal of response will be driven by the high individual propensity and not by the active TEs.

In this paper, we propose a novel methodology for evaluating the outcomes of a RCT in major depressive disorders (MDD) accounting for the predicted individual propensity probability. The principle is to use the estimated individual propensity to respond to placebo as a weight in the mixed-effect model for repeated measures (MMRM) analysis conducted to assess the TE. The TE is defined as the baseline-corrected change from placebo at study end. The higher is the individual probability of showing a placebo response, the lower will be the contribution of this subject in the assessment of TE. The expected effect of the MMRM weighed analysis will be to enhance the ability to detect a therapeutic signal as the contribution of subjects with high placebo responders will be minimized by the weighting procedure. The overall effect will be to enhance signal detection, with an increase of the effect size due to a better control of the inter-individual variability in the propensity to respond to placebo.

The estimation of the individual propensity probability to respond to placebo will be conducted using the Montgomery-Asberg Depression Rating Scale (MADRS) [21], or the 17-item Hamilton Depression Rating Scale (HAMD-17) [22] individual items change from screening to baseline in subjects assigned to treatment with placebo. A binary score will be associated with each subject: 0 or 1 for the absence or presence of a response at the study end. The predictive power of the individual item changes from screening to baseline to predict the response will be assessed using an artificial intelligence approach (AI).

Among the different methods used to implement AI, the multilayer perceptrons (MLP) artificial neural network (ANN) method has been shown to have superior and robust classification performance with respect to other methodologies, such as logistic analysis, random forest, and support vector machine [23]. The ANN predictive model developed with the placebo data will be applied to the individual item changes from screening to baseline of the subjects included in the other treatment arms. In this way, the individual predicted probability of PE will be associated to each subject included in the RCT. The inverse of this value will be used as a weight of each subject in the MMRM analysis conducted to assess the TE. The probability to become a placebo responder at study-end was then computed for all subjects included in the different treatment arms using the neural network predictive model outcomes applied to the individual pre-randomization data. However, assuming that the independent variable in the analysis (i.e., the change from baseline) is function either of the propensity to respond to a non-specific intervention or to the allocated treatment. In a clinical trial, longitudinal data are collected to study the effect of treatment (or intervention) over time. A key feature of longitudinal data is that the response variable (the clinical score) is measured more than once on each subject, and these repeated measurements are likely to be correlated. The primary efficacy endpoint are usually analyzed using MMRM analysis. The model included fixed-effect terms for baseline score, treatment, visit and with treatment-by-visit interaction as the independent variables. Therefore, the effect of treatment could be estimated by directly comparing outcomes between the treatment groups assuming that the independent variable (i.e., the change from baseline) represents the ‘true’ treatment (or intervention) effect.

## Methods

The propensity to respond to placebo was defined as a clinically relevant percent change from baseline in the MADRS or HAMD-17 total score, and therefore in the absence of any active treatment intervention. The relevant improvement was estimated by connecting the MADRS change scores to the clinician global impression-improvement (CGI-I) scale scores, using the equipercentile linking method. A CGI-I score of 3 (‘minimally improved’) corresponded to an average reduction from baseline in the total MADRS score of 24.5%, a CGI-I score of 2 (‘much improved’) corresponded to an average reduction of 52.5%; and a CGI-I score of 1 (‘very much improved’) to an average reduction of 82% [24]. For the purpose of the present analysis, the percent change from baseline in MADRS scale used for assessing the placebo response was 38%: the median value between minimally and much improved CGI-I. Using the equipercentile linking method, it was identified the percent reduction in the HAMD-17 scale of 41% as the equivalent percent reduction of 38% in the MADRS scale [25].

A case study is presented using the data of the study 29060/810. Details on this study have been previously reported [9]. This was a randomized, double-blind, parallel-group, placebo-controlled study evaluating efficacy and safety of paroxetine controlled release (12.5 and 25 mg/day) versus placebo in patients with major depressive disorder conducted in 40 centers in the United States. The study protocol, any amendments, the informed consent, and other information that required pre-approval were reviewed and approved by a national, regional, or investigational center ethics committee or institutional review board. This study was conducted in accordance with “good clinical practice” (GCP) and all applicable regulatory requirements, including, where applicable, the 1996 version of the Declaration of Helsinki. Written informed consent was obtained from each subject prior to the performance of any study-specific procedures. Electronic case report forms (eCRFs) were provided for each subject’s data to be recorded.

The propensity weighted analysis was conducted using a 5-step approach:

Step 1: The pre-randomization (i.e., screening and baseline) and end of study data (EOS) (i.e., visit at 8 weeks) in subjects randomized to placebo were selected.

Step 2: A predictive model was developed to estimate the probability to be placebo responder after 8 weeks of treatment using ANN and data collected in the pre-randomization period.

Step 3: The model developed in step 2 was validated by comparing the model predicted probability to the observed placebo response.

Step 4: The ANN model developed in step 2 was used to predict the individual probability to be placebo responder using the pre-randomization data of all subjects randomized in the study (i.e., subjects in the different treatment arms).

Step 5: The inverse individual probability was used as a weighting factor in the MMRM analysis conducted on the longitudinal clinical scores to estimate the TE.

The procedure used for model development and validation was based on a generally accepted procedure. This procedure consists of the random split of the original dataset into three datasets:

1. The training set, applied for the ANN model development (in our case this dataset included 75% of the data in the placebo arm randomly selected).
2. The validation set, applied for an unbiased model evaluation. The evaluation was conducted by comparing the model predictions based on the model developed in point 1 with the data observed in the validation dataset (in our case this dataset included the 25% data in the placebo arm not used for model development).
3. The working dataset, with the full data set including all the subject data in the 3-arms. This dataset was used to provide the individual estimate of the propensity probability applying the ANN model validated in step 2 to the pre-randomization data of each subject in the 3-arms.

A binary score was associated to each subjects: 0 or 1 for the absence or presence of response after 8 weeks of treatment (i.e., HAMD-17 ≥ 38% or greater change). The ability of the early collected (between two pre-randomization time points at screening and baseline) HAMD-17 individual item to predict the response to placebo at week 8 was investigated using artificial intelligence (AI) methodology. The AI approach was selected as this methodology provides the most performing predictive tool today available [26]. Among the different methods used to implement AI, the ANN method was shown to have superior and robust classification performance with respect to other methodologies [23]. Artificial neural networks are computational brain-inspired systems which are intended to replicate the way that humans learn. Neural networks consist in at least of three layers of nodes: an input layer, at least one hidden layer and an output layer. Except for the input nodes, each node is expected to emulate the function of a neuron that uses a nonlinear activation function. ANN utilizes a supervised learning technique called backpropagation for training [27, 28]. The implementation of ANN requires the definition of two hyperparameters that control the topology of the network: the number of hidden layers and the number of nodes in each hidden layer. A grid search was initially conducted for identifying the optimal number of layers (i.e., 1, 2, or 3) and the optimal number of nodes (i.e., from 1 to 17) in an ANN model. Then, a bootstrap analysis was conducted on the best performing model to evaluate the predictive performance and the robustness of this model (i.e., the area under the receiver operating characteristic (ROC) curve, with the 95% confidence interval). The ANN analysis was conducted using the ‘*neuralnet*’ library in R [29]. The ANN model developed using only placebo data was used to predict the individual propensity to respond to placebo in each subject included in the three arms of the study.

The inverse of the estimated probability was included as weight in MMRM model used to analyze the longitudinal HAMD-17 total score and to assess the TE. The MMRM model was implemented in SAS (PROC MIXED, Version 9.4, SAS Institute, Carry, NC, USA), using the change from baseline of the HAMD-17 total score. In the MMRM analysis a random effect model was used on the change from baseline value, using an unstructured covariance matrix, time as a classification variable, and baseline measurement as a covariate, baseline × time interaction, and treatment × time interaction. A significance level of α = 0.05 was used to establish the significance of the TE.

Drug-placebo TE sizes were calculated as the least squares means (LS means) difference divided by the pooled standard deviation, obtained as the standard error of the LS mean difference divided by the square root of the sum of inverse treatment group sample sizes.

## Results

A total of 459 subjects were included in the test trial 810. Among them 58% were females and 42% males. The description of the demographic data of the MDD population is presented in Table 1.

**Table 1:** Demographic data of the MDD population.

Treatment	*N*	Variable	Mean	Std Error	Median	Min	Max
Paroxetine CR 12.5 MG	156	Age (year)	38.37	0.98	37.5	18	74
		Weight (kg)	83.55	1.75	80.97	45.8	165.2
		Day*	7.48	0.16	7	4	24
Paroxetine CR 25 MG	154	Age (year)	39.28	0.88	38.5	18	71
		Weight (kg)	83.98	1.69	80.51	51.5	146.5
		Day*	7.4	0.14	7	4	15
Placebo	149	Age (year)	38.64	0.97	37	18	65
		Weight (kg)	86.03	2.1	83.91	42.4	204.1
		Day*	7.76	0.18	7	4	17

*Days between screening and baseline visits.

The means (±SD) baseline total HAMD-17total score were 23.13 (±2.89), 23.51(±3.28), and 23.81 (±3.23) for paroxetine CR 12.5 mg, paroxetine CR 25 mg, and placebo, respectively.

The grid search analysis indicated that the optimal number of layers was 3 and the optimal number of nodes per layer was 12, 6, and 5, respectively. The optimality criteria was based on the best predictive performance of the model.

**Fig. 1:**
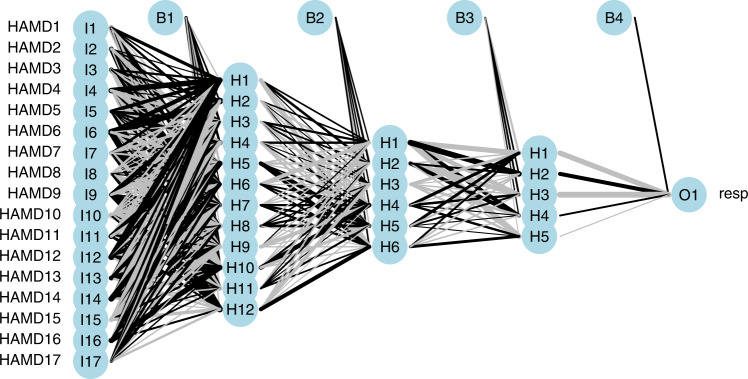
ANN Analysis. Final neural network layouts for the analysis conducted using the changes from screening to baseline of the individual items of the HAMD-17 clinical scale used as potential predictors of the response (resp = response to placebo).

The final neural network layout for the ANN analysis is presented in Fig. 1. In this plot, the first column represents the change from screening to baseline of the 17 individual items of the HAMD-17 scale considered as predictors of the placebo response (‘resp’), the second column represents the 12 combined items characterizing the first layer, the third column represents the 6 combined items defining the second layer, and the third column represents the 5 combined items defining the final layer. The lines connecting the nodes are color-coded by sign (black increasing, and gray decreasing effect).

The size of the connecting lines in the neural network are analogous to the coefficients in a standard regression analysis. They determine the relative influence of information that is processed in the network. A null weight will be associated to variables not relevant for predictions. The overall predictive performance of the ANN model was assessed using the area under the ROC curve (AUC). The value of the AUC was 0.81, with a 95% confidence interval of 0.64–0.97. This value, statistically greater than the noninformative threshold of 0.5, represents the predictive performance of the ANN model to predict the probability to show a non-specific response to a treatment using the individual item score changes of the HAMD-17 scale assessed in two pre-randomization time points (i.e., screening and baseline).

The ANN model was used to predict the individual propensity to respond to placebo in each subject included in the three arms of the study.

**Fig. 2:**
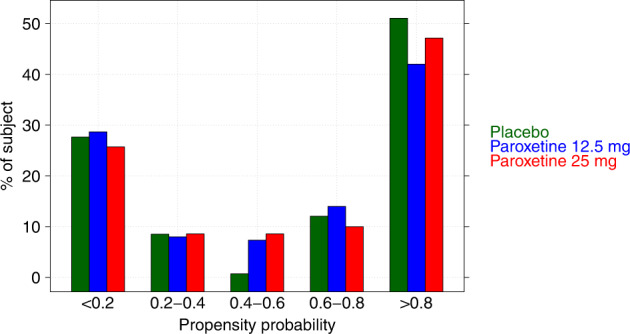
Propensity probability. Distribution of the propensity probability to a placebo effect by treatment.

The percentage of subjects with estimated propensity to respond to non-specific TEs in the intervals <0.2, 0.2–0.4, 0.4–0.6, 0.6–0.8, and >0.8 is presented in Fig. 2. The distribution of the propensity indicated that a large majority of the subjects have a high (>0.8) probability to inflate the response due to a non-specific response to a treatment. Therefore, the size of the TE is expected to be larger when the weighting factor will be included in the mixed-effect analysis to account for this unbalance.

**Fig. 3:**
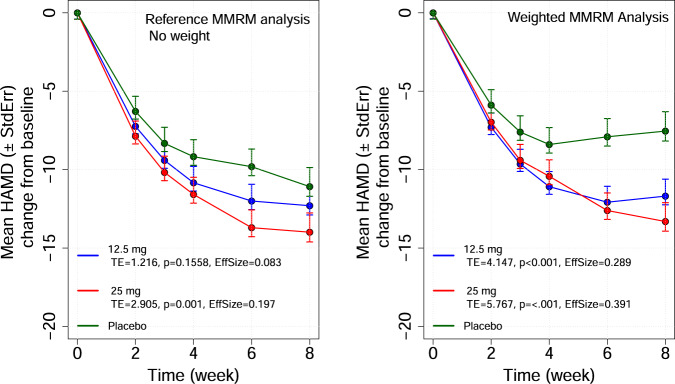
MMRM longitudinal analysis. Results of the non-weighted and weighted MMRM analyses with the estimation of the effect sizes. The LS mean (± standard error) of the longitudinal HAMD-17 total score changes from baseline are presented by treatment.

The results of the non-weighted and weighted MMRM analyses with the estimation of the effect sizes are presented in Fig. 3. Note that by definition, the results of the reference analysis will be the same in absence of weight or in presence of a weight identical for each subject.

A sensitivity analysis was conducted to evaluate the impact of the excessively high and excessively low propensity to a PE on the estimated TE with and without the use of a propensity weight in the MMRM analysis. Three analyses were conducted with and without propensity weight by (i) removing the subjects with high probability of a PE (Prob > 0.8), (ii) removing the subjects with very low probability of a PE (Prob < 0.1), and (iii) including all subjects. The results of the analyses are presented in Table 2.

**Table 2:** Sensitivity analysis results to evaluate the impact of the excessively high and excessively low propensity to a placebo effect on the estimated TE with and without a propensity weigh in the MMRM analysis.

Analysis	Comparison	TE	*P*	Effect-size
Propensity Weight	12.5mg_vs_Plac	−4.147	<0.0001	0.289
	25mg_vs_Plac	−5.767	<0.0001	0.391
No data with prob < 0.2	12.5mg_vs_Plac	−2.154	0.043	0.119
	25mg_vs_Plac	−5.951	<0.0001	0.329
No data with prob > 0.8	12.5mg_vs_Plac	−4.533	<0.0001	0.231
	25mg_vs_Plac	−6.067	<0.0001	0.299
No Propensity Weight	12.5mg_vs_Plac	−1.216	0.1558	0.083
	25mg_vs_Plac	−2.905	0.0011	0.197
No data with prob < 0.2	12.5mg_vs_Plac	0.092	0.9258	0.005
	25mg_vs_Plac	−2.103	0.0371	0.125
No data with prob > 0.8	12.5mg_vs_Plac	−3.791	0.0018	0.185
	25mg_vs_Plac	−5.960	<0.0001	0.281

The analysis with and without propensity weight indicated that the weighted analysis provided an estimate of TE and an effect-size about twice larger than the non-weighted analysis. In this analysis: (i) the TE increased when the subject with high probability of a PE were removed, and (ii) the TE decreased when the subject with low probability of a PE were removed. These findings are consistent with the expected effect of low/high placebo response on the estimated/estimable TE.

**Fig. 4:**
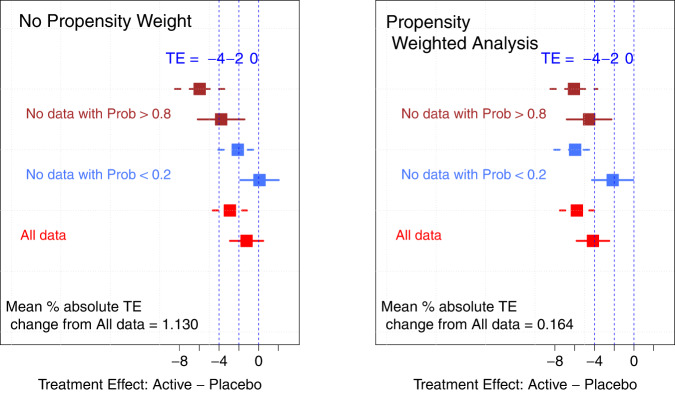
Sensitivity analysis. Propensity weighed and non-weighted analyses: comparison of the estimated TE in the total population (All data) and in population without high (Prob  >  0.8) and without low (Prob  <  0.2) placebo response. The dots represent the TE value estimated in the MMRM analysis, the horizontal lines represent the 95% confidence intervals (the solid lines correspond to the 12.5 mg arm and the dotted lines corresponds to the 25 mg arm). The vertical blue dotted lines represent some reference TE values of −4, −2, and 0.

The % absolute deviation from the TE (i.e., bias) estimated in the total population (all data) and in population without subjects with high (Prob > 0.8) and with low (Prob < 0.2) probability of a PE was estimated and compared in the propensity weighted and non-weighted analyses (Fig. 4).

The estimated % absolute deviation of the TE values was 1.13 and 0.164 for the conventional and the propensity weighted analyses, respectively. This large difference indicates that the propensity analysis is less sensitive to the presence of excessively low or excessively high placebo responders due to the effect of the weight probability. On the contrary, the estimated TE in the conventional MMRM analysis was significantly influenced by the distribution of the different level of placebo responders and non-responders.

## Discussion

In drug development, usually researchers want to compare two medications to understand which one is more effective in treating or preventing disease. Randomized controlled trial is widely accepted as the best design for evaluating the efficacy of a new treatment because the randomization is expected to eliminate accidental bias, including selection bias, and to provide a base for a fair comparison of the TE.

The TE in clinical trials for MDD is usually considered as the resultant of treatment specific and non-specific effects. The baseline individual propensity to respond to any treatment is considered as a major non-specific confounding factor. The larger is the baseline propensity to respond to non-specific TEs the lower is the chance to detect any treatment specific effect. In the current clinical trial setting no methodologies are currently available for evaluating the comparability of the treatment arm with respect to the potential baseline unbalance in the distribution of the individual propensity to respond to placebo.

To address comparability issues among groups, epidemiologists have developed specific methodologies which include propensity score matching and weighting, focused on creating baseline comparability between the treatment groups corrected by potential confounding factors. The propensity score methodology was initially developed for mitigating the confounding bias in non-randomized comparative studies and to facilitate causal inference for TEs [30].

This methodology was used mainly in epidemiological and social science studies, until it was adopted in a regulatory setting by statisticians in FDA/CDRH, where it was used in observational studies that supported marketing applications for medical devices [31, 32]. Since 2018, the scope of the propensity score methodology has been broadened so that it can be used for the purpose of leveraging external data to augment a single-arm or randomized traditional clinical study [33].

Regulatory agencies are well aware of the relevance of the propensity weighting methodology for insuring comparability of treatment arms, mainly in the analysis of observational studies [34, 35]. On this basis, we believe that there are valid methodological reasons for the regulatory agencies to consider the extension of the propensity methodology in RCTs in CNS as a reference analysis suitable to control the unknown potential baseline unbalance in the distribution of the propensity to non-specific placebo response.

The methodology developed in this paper assumes that the effect of a treatment in a major depressive disorder (MDD) trial can be viewed as the resultant of treatment-specific and treatment non-specific effects. While the specific effect can be associated with the active drug response, the non-specific effect can be attributed to a generic individual propensity to respond to any treatment or intervention. As we have previously described [36], one may classify treated patients in an MDD trial based on each participant’s propensity to respond to a given type of treatment. The “D − P − ” population comprises patients who are not responsive to either active treatment (D) or placebo treatment (P), whereas the “D + P − ” population comprises patients who are responsive to active treatment but not to placebo. The “D + P + ” population comprises patients who are responsive to either active (D) or placebo (P) treatments, and are therefore uninformative, given their propensity to respond to non-specific TEs. The propensity can be considered as a major non-specific prognostic and confounding effect. The larger is the baseline propensity to respond to placebo, the lower will be the chance to detect any treatment specific effect [14]. The statistical methodologies currently applied for analyzing RCTs do not account for potential unbalance in the allocation of subjects to the treatment arms associated with different distribution in the individual propensity to respond to placebo. Hence, the groups to be compared may be imbalanced, and thus incomparable due to baseline differences.

The basic premise of the proposed methodology is that the changes in the individual items of a clinical scale used for the assessment of the disease severity collected between screening and baseline visits prior to the treatment allocation contains relevant information of the time course of the disease, as reported by Hopkins et al. using the PANSS score [37]. The response to placebo was defined as a clinically relevant percent change from baseline in the MADRS or HAMD-17 total score (i.e., a reduction of at least 38% and 41%, respectively). The relevant improvement was estimated by connecting MADRS to CGI-I scales using the equipercentile linking method and by selecting the percentage reduction associated with minimal and much improved CGI-I score.

An ANN analysis was conducted to evaluate the predictive performances of the individual item values of the target clinical scale (i.e., MADRS and HAMD) evaluated in the same subject in two pre-randomization time points (i.e., screening and baseline visits) in subjects treated with placebo. The ANN model was then applied to the pre-randomization data of all subjects in the trial to associate to each subject a probability score representing the individual propensity to respond to placebo. This individual score was then used as a propensity weighting factor in the MMRM analysis conducted for assessing the TE to reduce baseline imbalances between arms.

A case study was presented using the data of a randomized, double-blind, placebo controlled, three arms, parallel group, 8 weeks duration, fixed-dose study to evaluate the clinical efficacy of paroxetine CR at the doses of 12.5 and 25 mg/day. This ANN model performed satisfactorily well in terms of predictive performance estimated by the area under the ROC curve of 0.81. This model was used to predict the individual propensity probability to respond to placebo in each subject included in the three arms. The distribution of the propensity probability in the different treatment arms indicated a large unbalance in the distribution of the high probability values (i.e., > 0.8).

The inverse of the estimated probability was included as weight in the mixed-effects model for the repeated measures model used to assess the TE. The comparison of the results of the analysis with and without the propensity weight indicated that the weighted analysis accounted for the individual probability to respond to placebo and provided an estimate of the TE (difference in the change from baseline between placebo and active at week 8) and of the effect-size about twice larger than the conventional non weighted analysis. The resulting effect of the inclusion of the estimated probability to be placebo responder as a weighting factor in the analysis was to provide an estimate of the TE adjusted for the difference in the individual propensity to respond to placebo and to better control the impact of subjects with high placebo response.

The results presented indicated that the individual weights obtained in one RCT cannot be generalized and prospectively used in other trials even if the other trial has a similar design. This is because the propensity weight represents a subject-specific attribute varying from individual to individual. Therefore, as the subjects enrolled in different trials are different, the weights obtained in one trial cannot be prospectively used in another trial.

According to the FDA definition, enrichment is the prospective use of any patient characteristic to select a study population in which detection of a drug effect (if one is in fact present) is more likely than it would be in an unselected population [38]. Therefore, the propensity weighting approach cannot be considered as a population enrichment method because all the randomized subjects are included in the analysis. Prospectively, the propensity weighted analysis can be applied to any current phase II, phase III, or historical RCTs when the following conditions are satisfied: (i) the study has been designed to collect screening and pre-treatment baseline data, (ii) the criteria for assessing the clinical response to placebo has been pre-specified in the statistical analysis plan (SAP), (iii) the acceptable criteria for the predictive performance of the ANN model used to estimate the link between screening and baseline data to the placebo response has been also pre-specified in the SAP specifying that the ROC AUC cut-offs should be statistically greater than the noninformative threshold of 0.5.

The benefit of this approach in phase II is to dispose a tool for a more precise and conservative estimate of the TE adjusted by possible excessively low or excessively high level of placebo response as shown by the results of the sensitivity analysis. The estimated bias in the assessment of the TE due to the presence of very high and very low placebo responders using the conventional and the propensity weighted analysis indicates that the propensity analysis is less sensitive to the presence of excessively low or excessively high placebo responders due to the effect of the weight probability. On the contrary, the estimated TE in the conventional MMRM analysis was significantly influenced by the distribution of the different level of placebo responders and non-responders.

Historical attempts to identify and deal with placebo responders were based on innovative study design aimed to identify and exclude high placebo responders. Among these study designs, we can mention the lead-in periods [6] or the sequential parallel comparative design [7]. In addition, alternative analysis procedures such as the band-pass methodology were proposed for detecting and removing recruitment sites with non-plausible placebo response from the analysis.

The major difference and advantage of the proposed methodology with respect to the historical study design and/or analysis procedures is that no subject will be excluded and all subjects randomized in the study will be included in the analyses. The propensity weighting method provides an unbiased strategy to associate the observations collected in each subject with a weight accounting for the potential confounding factor of a non-specific response. The comparison of the results of the analysis with and without the propensity weight indicated that the weighted analysis accounted for the individual probability to respond to placebo and provided an estimate of the TE (difference in the change from baseline between placebo and active at week 8) and of the effect-size about twice larger than the conventional non-weighted analysis. The resulting effect of the inclusion of the estimated probability to be placebo responder as a weighting factor in the analysis was to provide an estimate of the TE adjusted for the difference in the individual propensity to respond to placebo and to better control the impact of subjects with high placebo response. Despite the relatively large size of the clinical study considered, the main limitation of this study is the restricted number of RCTs evaluated with the proposed methodology, even though similar results have been found in the analysis of additional RTCs not reported in this paper. Finally, we do not identify scenarios where the use of the propensity methodology would not be appropriate, of course, when the applicability criteria are satisfied.
